# “Beneficial Microbes: Food, Mood and Beyond”—Editorial and the Perspectives of Research

**DOI:** 10.3390/microorganisms11041014

**Published:** 2023-04-13

**Authors:** Mohamed Zommiti, Mounir Ferchichi, Marc G. J. Feuilloley

**Affiliations:** 1 Research Unit Bacterial Communication and Anti-infectious Strategies (CBSA, UR4312), University of Rouen Normandie, 27000 Evreux, France; 2 Normandie University, UNICAEN, UNIROUEN, ABTE, 14000 Caen, France; 3 Unité de Protéomique Fonctionnelle et Potentiel Nutraceutique de la Biodiversité de Tunisie, Institut Supérieur des Sciences Biologiques Appliquées de Tunis, Université Tunis El-Manar, Tunis 1006, Tunisia

Amongst the list of beneficial microbes, lactic acid bacteria (LAB), *Bifidobacterium* sp., probiotics, *Bacillus* sp., and even several yeasts seem to be promising alternatives with which to fight chemicals, synthetic pharmaceuticals, diverse ailments, and antibiotic resistance. Within this line of research, probiotics, with their relatives and bioactive metabolites, including pre-, pro-, post-, para-, and psychobiotics, seem to be the salient groups of bacteria and bioingredients that have positively impacted animal and human life for centuries, and still do currently [1,2,3]. Different benefactions have been attributed to probiotic consumption in clinical trials as well as in vitro and in vivo experimental models. In this regard, probiotics have been broadly used for the treatment of various diseases, including metabolic disorders, aging, inflammation, cancer, obesity, hypertension, diabetes, antivirals, anxiety, depression, and so on [4,5,6,7]. Similarly, bacteriocins, as the most important metabolites produced by probiotics and as novel therapeutic treatments, have shown relevant evidence in terms of fighting pathogens, multifaceted antimicrobials, and anticancer activity, reflecting wide applications in the fields of the food industry, agriculture, and veterinary as well as human health [7].

Emotional wellbeing has a direct impact on physical wellbeing and vice versa. Regarding the two-way crosstalk between the brain and the gastrointestinal system, it has been unveiled that this axis is firmly mediated by gut bacteria [8]. These bacteria, being in eubiosis or in dysbiosis, produce neurochemicals and interact with neurons present in the enteric nervous system. Several representatives of gut microbiota and/or beneficial microbes have the ability to produce or stimulate the production of neurotransmitters, short-chain fatty acids (SCFAs), enteroendocrine hormones, and anti-inflammatory cytokines. Owing to such potential, these specific microorganisms, also known as “psychobiotics”, showed astonishing applications, ranging from mood and stress alleviation to being an adjuvant in therapeutic treatment for various neurodevelopment and neurodegenerative disorders [9].

This Special Issue, on “Beneficial Microbes: Food, Mood and Beyond”, sheds light on the world of beneficial microorganisms, encompassing LAB, *Bifidobacterium* sp., *Bacillus* sp., some yeasts, and probiotics as well as their bioactive metabolites, a continuous story of success in various fields, in particular fighting sturdy pathogens, promoting health, boosting the immune system, treating diseases, and even prolonging the lifespans of humans as well as animals. It contains three research articles, one communication paper, and one review, discussing the importance of using probiotics and their relevant contributions to food, mood, and other related fields. To begin with, the experimental paper produced by Vasiliauskaite et al. [10] examined the antifungal potential of Lactobacilli strains isolated from fermented cow milk in order to select strains with relevant fungicidal properties and develop bioprotective acid whey protein concentrate (AWPC)-based fermentates. The findings unveiled the selection of the *Lacticaseibacillus paracasei* A11 AWPC fermentate and its use as a vehicle for protective culturing in the development of a pectin-AWPC-based edible coating. The research paper by Chen et al. [11] revealed the identification of six novel genes that can be used as targets to design primers for the rapid screening of bile-salt-tolerant *lactobacilli*. Overall, the obtained results deepen our understanding of bile salt tolerance mechanisms in *Lactobacillus* and provide a basis for further rapid assessments of tolerant strains. Within the same framework, Smith and collaborators [12] investigated the effect of a 10-week resistance training regimen with or without peanut protein supplementation in untrained young adults on fecal microbiota and mood disturbance. The main findings revealed significant changes in microbial taxa, particularly an increase in microbial diversity following 10 weeks of resistance training, reflecting an intricate relationship between mood disturbance and body composition vicissitudes. Regarding the communication published by Adnan and Chapwanya [13], it deeply examined the implication of endometrial microbiome alteration on endometrial inflammation, ovarian activity, fecundation, pregnancy, and postpartum complications. Additionally, it unveiled the crucial role played by the administration of probiotics at an intravaginal scale in cattle, to substitute antibiotic and hormone therapy in order to treat uterine disease, thereby underscoring the importance of probiotics as a live biotherapeutic that is not only for humans; however, the review paper by Thangaleela et al. [14] investigated the role of probiotics and diet in the management of neurological diseases and mood states. It reported significant evidence for the salient role of probiotics in solo or combined with diet in neuroprotection and managing representative neurological disorders, injuries, and mood states. This work has emphasized the bidirectional crosstalk between gut microbiota and the brain, designated as the “microbiota–gut–brain axis”, opening the gate in front of scientific investigation to consider the gut microbiome as a novel target that can improve mood and mental health.

To wrap up, a crucial question must be asked: why do we feel the need to use the term next-generation probiotics (NGPs)? We believe that, in the near future, the nonstop investigative works and research on the beneficial impact of the microbiome on human health will lead to the discovery and development of novel microorganisms derived from our microbial symbionts. Such microbes may likely belong to uncommon and formerly uncharacterized microorganisms with unusual properties, or perhaps may even be microorganisms formerly thought of as pathogens or pathobionts. These advancements will cause relevant challenges for scientific investigation, industrial exploitation, and regulatory agencies. Currently, the term NGPs can serve as a useful descriptor for these “non-conventional” microorganisms. Within the same context, other human commensals developed as well as approved through a pharmaceutical pathway and that revealed a high potential to cure ailments and/or alleviate symptoms will probably retain the live biotherapeutic product (LBP) moniker.

The term probiotic is not a taxonomic one, but refers to functionality. Modulating microbiota by using probiotics or next-generation beneficial microbes represents a future perspective for the development of either nutritional and/or pharmaceutical approaches to maintain human wellbeing and animal welfare. Nevertheless, the implementation of a typical series of pharmaceutical clinical trials is urgently needed to translate research into clinical practice, to refine the clinical indication of specific probiotic strains, and to better understand the postbiotic effect of substances released by probiotic bacteria as well as the parabiotic effect of inactivated bacterial cells [15]. Currently, the widespread nature of various ailments, the non-efficiency of a wide range of pharmaceuticals, and the upsurge in critical multidrug resistance seem to be a heavy burden on health systems around the globe. All of the aforementioned indicators point to the absence of any escape from the ESKAPE pathogens. Additionally, the nonstop advancements in nutraceuticals, probiotic formulations, LAB bioengineering, and next-generation probiotics, notably live biotherapeutics, are giving birth to promising alternatives and tools in combatting sturdy pathogens, their antibiotic resistance, and the severe dysbiosis as well as ailments that they may cause.
